# Influenza outbreak management tabletop exercise for congregate living settings

**DOI:** 10.1017/ash.2023.528

**Published:** 2024-01-11

**Authors:** Christina K. Chan, Jerome A. Leis, Heather Candon, Neethu R. Thomas, Jaclyn O’Brien, James Callahan, Brigitte Pascual, Marianna Ofner, Fatima Fazalullasha, Adrienne K. Chan, Jeff E. Powis, Charlie Tan

**Affiliations:** 1 Infection Prevention and Control, Sunnybrook Health Sciences Centre, Toronto, ON, Canada; 2 Division of Infectious Diseases, Department of Medicine, University of Toronto, Toronto, ON, Canada; 3 Infection Prevention and Control, Toronto East Health Network, Toronto, ON, Canada; 4 Toronto Public Health, Toronto, ON, Canada

## Abstract

We conducted a tabletop exercise on influenza outbreak preparedness that engaged a large group of congregate living settings (CLS), with improvements in self-reported knowledge and readiness. This proactive approach to responding to communicable disease threats has potential to build infection prevention and control capacity beyond COVID-19 in the CLS sector.

## Introduction

Outbreaks of respiratory viruses are common in congregate living settings (CLS), including long-term care homes (LTCHs) and retirement homes (RHs), and are associated with significant morbidity and mortality among residents.^1^ In Canada, seasonal influenza causes on average 12,200 hospitalizations and 3,500 deaths every year, with higher risk of poor outcomes among elderly persons.^1^ Residents of CLS are especially vulnerable; they are typically older with underlying health conditions and physical limitations, and the congregate setting increases risk of transmission.^1^

The CLS sector was greatly impacted by the COVID-19 pandemic, resulting in significant improvements to infection prevention and control (IPAC) practices, including surveillance, testing, masking and personal protective equipment (PPE), vaccination, and dedicating IPAC Leads within CLS.^2^ Despite this progress, IPAC-related capacity in CLS for managing non-COVID-19 respiratory viruses remains limited, with new IPAC professionals recruited during a pandemic period when other respiratory viruses were not co-circulating.^3^

Tabletop exercises are a practical and scalable method for evaluating and supporting situational readiness,^4^ and have been used to foster preparedness against communicable disease threats.^5,6^ We describe our experience in conducting a tabletop exercise on influenza outbreaks for CLS and assess the impact of the exercise on participants’ self-reported knowledge and readiness.

## Methods

### Development and design of tabletop exercise

The North Toronto and East Toronto IPAC Hubs have supported 33 LTCHs, RHs, and other CLS since October 2020. In October 2022, both hubs partnered with the local public health unit to conduct and evaluate an influenza preparedness tabletop exercise for all supported CLS. The objective was to practice investigation and management of an influenza outbreak in a simulated setting, promoting preparedness for the upcoming influenza season. The exercise was tailored specifically to staff working in CLS and included guideline recommendations for control of respiratory outbreaks in CLS,^7^ as well as lessons learned from real-world experience. Four knowledge domains were prioritized: (1) case definition and testing for influenza, (2) criteria to declare an influenza outbreak, (3) syndromic surveillance and reporting of cases, and (4) outbreak control measures, including case management, administrative controls, PPE, and treatment and chemoprophylaxis.

A 12-item pre- and post-exercise questionnaire was created for participants to measure self-reported change in the following constructs: knowledge of influenza case and outbreak management, preparedness for outbreak management, and perceived importance of CLS IPAC Leads in outbreak response. A five-point Likert scale ranging from 1 (strongly disagree) to 5 (strongly agree) was used to score each item. We also created an evaluation of the tabletop exercise to obtain feedback.

### Content of tabletop exercise and implementation

The tabletop exercise was a discussion-based exercise conducted on a virtual platform. Participants were initially presented with a scenario involving a suspected influenza outbreak in a LTCH, with two residents developing upper respiratory symptoms and testing positive for influenza A. Subsequently, two more symptomatic residents and one symptomatic staff member are identified. Participants were divided into small breakout groups, each led by facilitators from the IPAC Hubs. Representatives from the local public health unit provided content expertise. Participants were then brought together in a large group to summarize key lessons and debrief their experiences.

Participants were asked to complete the pre- and post-exercise questionnaires before and after the exercise, respectively.

### Data analysis

Descriptive analysis of categorical variables was performed by calculating frequencies and percentages. Paired median pre- and post-exercise questionnaire scores were calculated and compared using the Wilcoxon signed-rank test. *P*-value <0.05 was considered statistically significant. Statistical analyses were performed using STATA SE 18.0 (StataCorp, College Station, TX, USA). Research ethics review was not required because the study met criteria for exemption as a quality improvement project that was not human-subject research.

## Results

There were 52 participants in total, among whom 30 (57.7%) completed the pre- and post-exercise questionnaires. Participants were comprised of staff at LTCHs (40.0%, 12/30), other CLS (33.3%, 10/30), and RHs (26.7%, 8/30). The majority of participants worked at a facility with a dedicated IPAC Lead (66.7%, 20/30). Most had at least four to six years of working experience (73.3%, 22/30), but 50% (15/30) had no previous experience in managing non-COVID-19 respiratory outbreaks.

The median pre- and post-exercise questionnaire scores are shown in Table 1. There were statistically significant improvements in knowledge of case definitions of COVID-19 and non-COVID-19 respiratory viruses (4.0 vs. 5.0, *p* < 0.005), influenza antiviral treatment and chemoprophylaxis (4.0 vs. 5.0, *p* < 0.01), process for accessing antivirals (4.0 vs. 5.0, *p* < 0.001), staff return-to-work (4.0 vs. 5.0, *p* < 0.001), and readiness to manage respiratory outbreaks (4.0 vs. 5.0, *p* < 0.001).

**Table 1.** Median pre- and post-tabletop exercise questionnaire scores

Questions	Median (interquartile range)		*p*-value
	Pre-score	Post-score	
1. I understand the importance of vaccination against influenza.	5.0 (0)	5.0 (0)	0.25
2. I am confident about when to initiate droplet contact precautions on a resident showing respiratory symptoms, and when they should be discontinued.	5.0 (1.0)	5.0 (0)	0.13
3. I am confident about the isolation requirements for residents at my home exhibiting signs of influenza or influenza-like illness.	4.0 (1.0)	5.0 (1.0)	0.07
4. I am aware of the case and outbreak definitions of respiratory viruses (COVID-19 vs. influenza).	4.0 (1.0)	5.0 (1.0)	**<0.005**
5. I am confident about the treatment and chemoprophylaxis available for influenza.	4.0 (1.0)	5.0 (1.0)	**<0.01**
6. I am aware of the process of how to access antiviral treatment and chemoprophylaxis.	4.0 (1.0)	5.0 (1.0)	**<0.001**
7. I understand the procedure of reporting symptomatic cases to the IPAC Hub and local public health unit.	5.0 (1.0)	5.0 (0)	0.32
8. I understand staff policies for working on a floor/unit experiencing a respiratory outbreak.	4.5 (1.0)	5.0 (1.0)	0.06
9. I am aware staff can refuse to work/receive vaccination/receive prophylaxis.	5.0 (1.0)	5.0 (1.0)	0.19
10. I am aware of when staff can return to work following infection with influenza.	4.0 (1.0)	5.0 (1.0)	**<0.001**
11. I am prepared to manage a respiratory outbreak.	4.0 (1.0)	5.0 (0)	**<0.001**
12. I am aware of the process of declaring an influenza outbreak over.	5.0 (1.0)	5.0 (1.0)	0.05

Table 2 shows participants’ evaluations of the tabletop exercise. All participants agreed the scenario was plausible and realistic, the facilitators were knowledgeable, the exercise was appropriate for their respective positions, and participation enhanced readiness to manage respiratory outbreaks. Over 96% (29/30) of participants agreed the objectives were clear, they felt supported in their breakout groups, and the exercise provided the opportunity to address outbreak-related decisions related to their facilities’ critical mission areas. Selected participant comments included: “The format and facilitation in the breakout rooms was excellent,” “It was excellent and very informative. I enjoyed the interaction,” “[I enjoyed] how interactive it was,” “Very engaging,” and “[I enjoyed the] use [of] breakout rooms to ask specific questions.”

**Table 2.** Evaluation of tabletop exercise

Tabletop evaluation	Frequency (%)				
	Strongly Agree	Agree	Neutral	Disagree	Strongly Disagree
1. The objectives of the tabletop exercise were clear.	24 (80.0)	5 (16.7)	1 (3.3)	0 (0)	0 (0)
2. The tabletop exercise scenarios were plausible and realistic.	26 (86.7)	4 (13.3)	0 (0)	0 (0)	0 (0)
3. The facilitator(s) was knowledgeable about the material and kept the exercise on target.	21 (70.0)	9 (30.0)	0 (0)	0 (0)	0 (0)
4. Participation in the exercise was appropriate for someone in my position.	20 (66.7)	10 (33.3)	0 (0)	0 (0)	0 (0)
5. The breakout room exercise provided the opportunity for meaningful discussions.	23 (76.7)	4 (13.3)	3 (10.0)	0 (0)	0 (0)
6. I felt supported during the breakout room exercise with my peers and professional leads.	23 (76.7)	6 (20.0)	1 (3.3)	0 (0)	0 (0)
7. The exercise provided the opportunity to address significant decisions in support of critical mission areas	24 (80.0)	5 (16.7)	1 (3.3)	0 (0)	0 (0)
8. After this exercise, I am better prepared to deal with the capabilities for managing a respiratory outbreak.	25 (83.3)	5 (16.7)	0 (0)	0 (0)	0 (0)

## Discussion

In advance of the 2022–2023 respiratory virus season, we conducted a tabletop exercise to enhance influenza preparedness among partner CLS. Half the participants had no prior experience with management of influenza outbreaks, and pre- and post-exercise questionnaires demonstrated significant increases in their self-reported readiness in identifying outbreaks, administering antiviral treatment and chemoprophylaxis, applying staff return-to-work policy, and implementing outbreak control measures.

Although our study did not evaluate changes in practice related to the tabletop exercise, these key learnings addressed several critical components of influenza outbreak management that are associated with blunting transmission and reducing resident morbidity and mortality.^8–10^ In particular, delays in initiation of antiviral chemoprophylaxis are associated with heightened risk of transmission.^10^ This enhanced capacity equipped IPAC Leads across a whole healthcare sector before many had their first experience managing influenza in their facilities.

Tabletop exercises have been shown to be pragmatic and efficient tools for organizations to build capacity in responding to situational threats, identifying knowledge gaps, and enhancing preparedness.^4–6^ They also promote collaboration between colleagues and provide opportunity for cross-pollination with important partners. Our study adds to the literature by demonstrating the applicability of tabletop exercises to CLS, a sector where IPAC capacity remains limited and needs ongoing strengthening. We also included participants from various types of CLS and with differing levels of IPAC experience, showing their scalability to different audiences.

There are limitations to this study. The sample size was small with non-universal response rate, and only CLS in our specific region participated. These factors may limit the generalizability of our findings. It is also difficult to determine whether improvements in participants’ self-reported readiness reflect knowledge acquisition, as could have been evaluated with content-based questions, or enhanced influenza-related processes and outcomes. However, the improvements pertained to domains associated with reduced influenza transmission. Evaluating impact on patient/resident-level or facility-level outcomes is an important area for future research.

Our tabletop exercise helped improve identification and response to influenza outbreaks among CLS staff lacking significant experience in non-COVID respiratory outbreaks. This approach may be effective in building IPAC capacity in CLS beyond the COVID-19 pandemic.
